# Screening *Aspergillus flavus*, *Talaromyces purpureogenus*, and *Trichoderma koningiopsis* for Plant-Growth-Promoting Traits: A Study on Phosphate Solubilization, IAA Production, and Siderophore Synthesis

**DOI:** 10.3390/jof10120811

**Published:** 2024-11-22

**Authors:** Thabo J. Moropana, Elbert Lukas Jansen Van Rensburg, Livhuwani Makulana, Nkateko N. Phasha

**Affiliations:** Department of Biochemistry, Microbiology and Biotechnology, University of Limpopo, Private Bag X1106, Sovenga 0727, South Africa; 201911255@keyaka.ul.ac.za (T.J.M.); elbert.jansenvanrensburg@ul.ac.za (E.L.J.V.R.); livhuwani.makulana@ul.ac.za (L.M.)

**Keywords:** phosphate solubilization, plant growth promotion, biofertilizers, fungi

## Abstract

The global rise in population has led to an increased demand for food production, necessitating the adoption of sustainable agricultural practices. Traditional methods often rely on synthetic chemicals that negatively impact both human health and the environment. This study aimed to screen soil fungal strains for plant-growth-promoting traits, specifically focusing on their ability to solubilize phosphates, produce indole-3-acetic acid (IAA), and synthesize siderophores. Fungal strains were identified using rDNA sequencing of the ITS regions, and their growth-promoting abilities were assessed in vitro. *Aspergillus flavus* JKJ7, *Talaromyces purpureogenus* JKJ12, and *Trichoderma koningiopsis* JKJ18 exhibited varying degrees of phosphate solubilization, with *T. purpureogenus* JKJ12 solubilizing the highest amount of tricalcium phosphate (TCP), while *A. flavus* JKJ7 was the most effective in solubilizing phytic acid calcium salt (PCS). In terms of IAA production, *A. flavus* JKJ7 produced the highest auxin concentration (68.51 mg/L), followed by *T. koningiopsis* JKJ18 and *T. purpureogenus* JKJ12. Additionally, *A. flavus* JKJ7 produced the highest amount of siderophores (83.7%), indicating its potential for improving iron uptake in plants. Principal Component Analysis (PCA) revealed distinct functional capabilities among the strains, particularly in phosphate solubilization and IAA production, suggesting their complementary use in consortium formulations. These results indicate that these fungal strains possess significant plant-growth-promoting traits and could be used as bioinoculants for sustainable agriculture, either as single strains or in combination

## 1. Introduction

The rapid increase in the global population has significantly heightened the demand for food production, placing immense pressure on agricultural systems [1]. To meet these demands, crop agriculture has relied heavily on synthetic chemicals to amend soils, often at the cost of environmental and human health [2,3]. Phosphorus (P) is a crucial nutrient for plant growth, but in many soils, it exists primarily in non-bioavailable forms, making it inaccessible to plants [4]. Even after the application of chemical P fertilizers, much of the phosphorus becomes fixed to soil minerals, remaining unavailable for plant uptake [5]. Plant cells predominantly absorb phosphorus in the form of orthophosphates hydrogen phosphate (HPO_4_^²−^) and dihydrogen phosphate (H_2_PO_4_^−^), with H_2_PO_4_^−^ being more prevalent in acidic soils. While plants may occasionally take up other phosphorus forms, such instances are rare, and orthophosphates are the primary form available for root uptake [6]. Non-bioavailable organic P forms in the soil include inositol phosphate, phospholipids, and nucleic acids, while inorganic P forms include calcium phosphate, aluminum phosphate, and ferric phosphate [7]. The lack of available phosphorus severely limits plant root development and overall growth [8].

While it is well established that soil fungi have the ability to solubilize phosphorus and provide other key nutrients, gaps still exist in our understanding of the efficiency of different fungal strains and their practical applications in agriculture [9,10]. This study aims to address this gap by focusing on fungal strains with plant-growth-promoting traits, specifically targeting their phosphate solubilization capacity, indole-3-acetic acid (IAA) production, and siderophore synthesis. To assess the relative efficiency of these strains, a detailed statistical analysis, including Principal Component Analysis (PCA), will be applied to quantify differences among strains based on their ability to solubilize both organic and inorganic phosphorus and synthesize IAA and siderophores. Identifying the most potent fungal strains with scientific certainty will help bridge the gap between laboratory findings and practical agricultural applications.

Fungi have unique abilities that make them particularly effective in promoting plant growth. Unlike bacteria, which have been widely studied for their plant-growth-promoting traits, fungi form extensive mycelial networks that increase their surface area for nutrient absorption, enhancing their ability to solubilize phosphorus [11]. Moreover, fungal strains tend to retain their growth-promoting traits even after multiple rounds of sub-culturing, making them stable and reliable candidates for biofertilizer development [12,13]. This study, therefore, focuses on identifying fungal strains that can solubilize phosphate, produce IAA, and synthesize siderophores, traits critical for plant nutrient uptake and growth [14].

Phosphate-solubilizing microorganisms (PSM), including fungi, play a vital role in converting insoluble phosphorus into bioavailable forms, which plants can then use for various metabolic processes [15]. This process is facilitated by the production of low molecular weight organic acids, which either chelate phosphorus’s cationic partners or lower soil pH to solubilize phosphorus [16]. Additionally, fungi produce phosphatases such as phytase, which hydrolyze organic phosphates, further releasing phosphorus into forms that plants can absorb [17,18,19]. Fungal solubilization of phosphorus is particularly important in agricultural soils where phosphorus fixation limits crop productivity.

IAA, a key phytohormone produced by several fungi, also plays a significant role in plant development, including root elongation, cell division, and response to environmental stresses [20]. While auxin production is not novel, its importance in the context of fungal interactions with plants is noteworthy. Unlike bacteria, which have been extensively studied for IAA production, fungi offer distinct mechanisms for promoting root development and improving nutrient uptake, especially in challenging soil environments [21]. Understanding the differences in IAA production efficiency among fungal strains is crucial for selecting the most effective strains for use as biofertilizers.

Siderophores are another important trait produced by fungi that facilitate iron uptake in iron-deficient soils [22]. Iron is essential for various plant metabolic processes, yet its bioavailability is often limited in soils with high pH [23]. Fungi secrete siderophores, which form complexes with Fe^3+^, making it more accessible for plant absorption [24]. In addition to enhancing plant nutrition, siderophores act as a defense mechanism, helping plants outcompete phytopathogens for essential nutrients like iron [25]. This dual role highlights the potential of fungi as bioinoculants in sustainable agriculture, particularly in soils with nutrient limitations.

The rationale for conducting this study in a controlled laboratory setting rather than in the field is to establish a baseline understanding of these fungal mechanisms in isolation, free from the confounding variables present in natural ecosystems. By identifying and characterizing the plant-growth-promoting traits of these fungi under controlled conditions, and applying advanced statistical analyses such as PCA, this study aims to identify the most efficient fungal strains with scientific certainty. These results lay the groundwork for future greenhouse and field experiments to further explore the practical relevance of these fungal biofertilizers in enhancing plant growth and nutrient availability in agricultural systems.

Thus, this study aims to screen soil fungal strains for their plant-growth-promoting traits—phosphate solubilization, IAA production, and siderophore synthesis—and evaluate their potential as biofertilizers. The use of statistical tools will help distinguish the most potent strains, contributing to the development of sustainable biofertilizer solutions that can serve as alternatives to chemical fertilizers.

## 2. Materials and Methods

### 2.1. Isolation of Soil Fungi

#### 2.1.1. Sampling Site

Six soil samples were collected for this study. The sampling sites were selected based on their suitability for isolating plant-growth-promoting fungi. Five of the samples were obtained from different locations on the University of Limpopo campus (South Africa) in areas rich in decomposed plant material, an environment conducive to fungal growth. The sixth sample was taken from a garden in Polokwane, South Africa. The sampling sites were chosen specifically for their potential to harbor diverse fungal species involved in nutrient cycling and plant growth promotion. The selection was driven by the presence of abundant organic material, which increases the likelihood of isolating fungi with plant-growth-promoting traits.

#### 2.1.2. Isolation Method

From each soil sample, 1 g was suspended in 10 mL of sterile distilled water in an Erlenmeyer flask. The flasks containing the soil suspensions were incubated overnight in a shaker incubator at 150 rpm and 30 °C to allow for microbial resuspension. Following incubation, the suspensions were serially diluted up to 10^−7^ to decrease microbial density, facilitating the isolation of distinct colonies. Aliquots from each dilution were spread onto Yeast–Malt (YM) agar plates, which contained 20 g/L glucose, 3 g/L malt extract, 3 g/L yeast extract, 5 g/L peptone, 0.2 g/L chloramphenicol (to inhibit bacterial growth), and 15 g/L agar. The plates were incubated at 30 °C for 4 days, during which fungal growth was monitored daily. Fungal colonies with distinct morphological characteristics, such as colony shape, spore formation, and pigmentation, were selected for further analysis. These colonies were sub-cultured onto fresh YM agar plates to obtain pure cultures. Once isolated, the pure fungal strains were stored on YM agar plates at 4 °C for later use.

### 2.2. Fungal Strain Identification

The fungal isolates JKJ7, JKJ12, and JKJ18 were identified at Inqaba Biotechnical Industries (Pty) Ltd, Pretoria, South Africa. Strain identification was based on ITS DNA sequencing. Fungal DNA was isolated and purified following the manufacturer’s instructions in the ZR Fungal/Bacterial DNA MiniPrepTM Kit (Zymo Research, Pretoria, South Africa). For ITS DNA sequencing, the ITS1-5.8S-ITS2 region was amplified using PCR forward and reverse primers, ITS-1 (5′-TCC GTA GGT GAA CCT GCG G-3′) and ITS-4 (5′-TCC TCC GCT TAT TGA TAT GC-3′), respectively. The amplification was performed in 25 µL reactions using EconoTaq Plus Green Master Mix (Lucingen, Pretoria, South Africa). The PCR reaction started with an initial denaturation at 95 °C for 2 min, followed by 35 cycles of 95 °C for 30 s (subsequent denaturation), 50 °C for 30 s (annealing), 72 °C for 1 min, and completed by a final extension at 72 °C for 10 min. The final extension was followed by cooling at 4 °C. The received sequences were used for species identification by searching databases using http://www.ncbi.nlm.nih.gov/BLAST/ (accessed on 21 July 2022) [26]. The fungal strains were further identified by inferring a phylogenetic tree using a method described in [27].

### 2.3. Qualitative Estimation of Organic (Phytic Acid Calcium Salt) and Inorganic (Tricalcium Phosphate) Phosphate Solubilization

The three fungal strains *Aspergillus flavus* JKJ7, *Talaromyces purpureogenus* JKJ12, and *Trichoderma koningiopsis* JKJ18 were screened for the ability to solubilize both inorganic and organic forms of phosphate by inoculating the isolates on the National Botanical Research Institute’s Phosphate (NBRIP) agar plates. The media were supplemented with each insoluble form of phosphate separately to serve as sole phosphorus (P) sources. The inorganic form of phosphate was supplied as tricalcium phosphate (TCP), whereas the organic form was presented as phytic acid calcium salt (PCS) [28,29]. The control plates for each phosphorus source were inoculated with non-phosphate-solubilizing fungal strains, while un-inoculated plates served as additional controls for each phosphorus source. The media were composed of 10.0 g/L glucose, 0.5 g/L (NH_4_)_2_SO_4_, 0.3 g/L NaCl, 0.3 g/L KCl, 0.3 g/L MgSO_4_·7H_2_O, 0.03 g/L FeSO_4_·7H_2_O, 0.03 g/L MnSO_4_·4H_2_O, 15 g/L agar, and 5.0 g/L TCP/PCS at pH 6.5. The plates were incubated at 30 °C for 7 days. The growth of the fungal colonies and the development of clear halos suggested the colony solubilizes phosphate [30,31].

### 2.4. Quantitative Estimation of Phosphate Solubilization

The three fungal strains, *A. flavus* JKJ7, *T. purpureogenus* JKJ12, and *T. koningiopsis* JKJ18, were subjected to a quantitative test for both inorganic and organic forms of phosphate [28,29]. An agar block (1 cm) of the fungal isolates from YM agar was used to inoculate 50 mL NBRIP broth at pH 6.5 in a 100 mL Erlenmeyer flask. The broths were separately supplemented with TCP and PCS as the sole sources of insoluble P complex. The control flasks for each P source were not inoculated, and all the flasks were incubated at 30 °C for 6 days on a rotary shaker at 150 rpm. An aliquot of 300 µL was sampled daily from each flask for measuring solubilized phosphate. The samples were centrifuged at 10,000 rpm for 10 min to obtain cell-free supernatants. The soluble phosphate in the cell-free supernatants was measured using the ascorbic acid method described by [32], with some modifications. The color-developing reagent was mixed with the cell-free supernatant and incubated at 45 °C for 10 min and then at room temperature for 50 min to observe color development. The absorbance was measured at 820 nm, and solutions of K_2_HPO_4_ with known concentration were used as a standard.

### 2.5. Quantitative Screening for Indole Acetic Acid Production (IAA)

The fungal strains *A. flavus* JKJ7, *T. purpureogenus* JKJ12, and *T. koningiopsis* JKJ18 were screened for Indole-3-acetic acid (IAA) production using a method described by [33], with some modifications. In a 100 mL Erlenmeyer flask, up to 50 mL YM broth supplemented with 1 g/L of L-tryptophan was inoculated with the fungi under study and incubated in a shaker incubator at 30 °C and 150 rpm for 6 days. Up to 300 µL aliquots were sampled daily from each flask to measure IAA concentration. The concentrations of IAA produced were measured from a cell-free culture supernatant obtained after centrifugation. The cell-free culture supernatant was mixed with twice the amount of Salkowski solution and incubated in the dark at 40 °C for 30 min. The amount of IAA in the culture supernatant was measured spectrophotometrically at 530 nm, using solutions of known concentrations of IAA as standards to estimate the concentrations of the IAA produced.

### 2.6. Quantitative Screening for Siderophore Production

The studied fungal strains *A. flavus* JKJ7, *T. purpureogenus* JKJ12, and *T. koningiopsis* JKJ18 were screened for siderophore production using a method described by [24]. The fungal strains were cultured in 100 mL Erlenmeyer flasks, each with up to 50 mL vitamin-free media consisting of 20 g/L Glucose, 3.5 g/L (NH_4_)_2_SO_4_, 1.5 g/L L-asparagine, 0.02 g/L l-methionine, 0.010 g/L L-histidine, 1 g/L KH_2_PO_4_, 0.5 g/L MgSO_4_, and 0.5 g/L NaCl. The flasks were incubated in a shaking incubator at 30 °C and 150 rpm for 6 days. The produced siderophores in the cell-free culture supernatant were measured quantitatively by the chrome azurol S (CAS) assay. The cell-free culture was mixed with CAS solution with a ratio of 1:1 and incubated in the dark at 37 °C for 5 min. The OD was measured at 630 nm, and the percentage of siderophore units was calculated as described by [24].

### 2.7. Statistical Analysis

The experiments were conducted in triplicate, with the numerical values represented as the mean ± standard deviation (SD) of the three replicates. Graphs were generated using Microsoft Excel, while Principal Component Analysis (PCA) was performed using Minitab v21.2.0.0 statistical software.

## 3. Results

### 3.1. Molecular Identification of Soil Fungi

**Figure 1:**
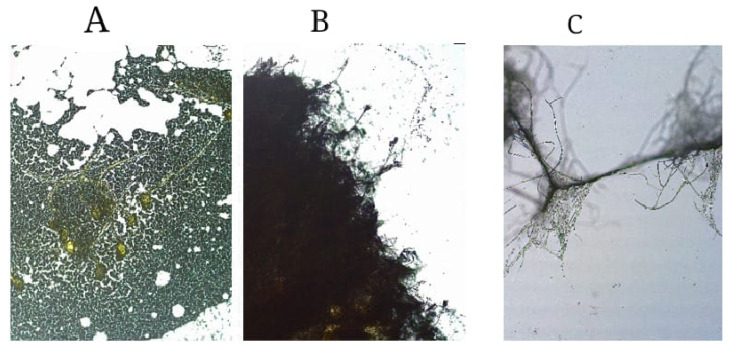
Images of the three P-solubilizing fungal isolates grown on YM agar media. The fungal strain *A. flavus* JKJ7 is represented by image (**A**), *T. purpureogenus* JKJ12 is represented by image (**B**), and *T. koningiopsis* JKJ18 is represented by image (**C**).

A total of 38 soil fungi were initially isolated, but a qualitative assessment of their phosphate solubilization abilities reduced this number to 18. These 18 fungal isolates were labeled JKJ1 to JKJ18. From this group, three isolates—JKJ7, JKJ12, and JKJ18—were selected for further study based on their superior phosphate solubilization performance in the qualitative analysis. These three fungal isolates, derived from a fungal library of soil fungi collected from the undersoil of decomposed plant matter, were identified and chosen for their potential plant-growth-promoting traits. Microscopic examination (magnified 40×) of the isolates revealed distinct differences in pigmentation, sporulation, and hyphal formation (Figure 1). Isolate JKJ7 (Figure 1A) displayed yellow-green pigmented spores and hyphae, while isolate JKJ12 (Figure 1B) exhibited dark green pigmentation. In contrast, isolate JKJ18 (Figure 1C) showed white to light yellow hyphal pigmentation, highlighting its distinct morphology compared with the other two isolates.

The conclusive revelation of their identities was made after comparing the sequences of their ITS region with those that already exist in the NCBI database. The fungal isolate JKJ7 showed 100% similarity with *Aspergillus flavus*, JKJ12 100% similarity with *Talaromyces purpureogenus*, and JKJ18 99.66% similarity with *Trichoderma koningiopsis* (Table 1).

### 3.2. Phylogenetic Tree Inferred for Fungal Strain Identification

**Figure 2:**
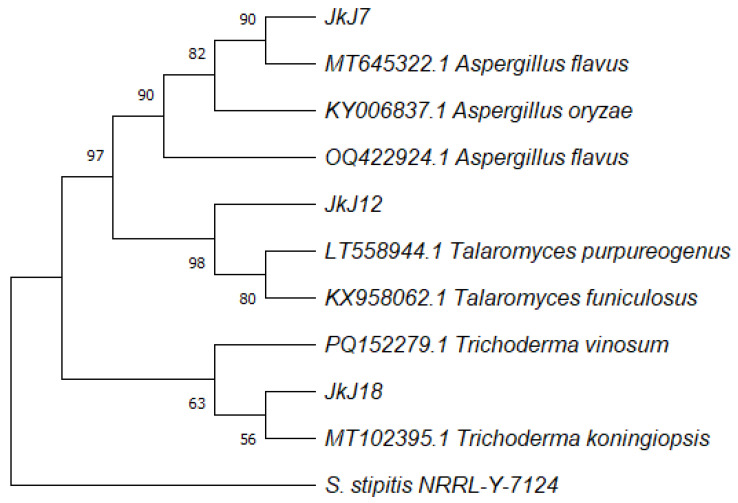
Neighbor-joining tree deduced using the ITS sequence of the three fungal strains isolated from the soil with reference fungal strains from NCBI. Only branches with more than 50% bootstrap support are shown. *S. stipitis* NRRL-Y-7124 was used as the out-group.

JKJ7 clusters closely with *Aspergillus flavus* (MT645322.1) and *Aspergillus oryzae* (KY006837.1). This is supported by a high bootstrap value (86%), indicating strong confidence in this grouping. JKJ7 is likely an *Aspergillus flavus* strain due to its close relationship with other *Aspergillus flavus* sequences, supported by the phylogenetic analysis. JKJ12 clusters closely with *Talaromyces purpureogenus* (LT558944.1) and is well separated from other species. The node linking JKJ12 and *Talaromyces purpureogenus* is strongly supported by a bootstrap value of 98%, indicating a reliable grouping. JKJ12 is likely a strain of *Talaromyces purpureogenus* based on its high bootstrap support and close phylogenetic relationship with the known strain. JKJ18 Identification: JKJ18 clusters closely with *Trichoderma vinosum* (PQ152279.1) and *Trichoderma koningiopsis* (MT102395.1), forming a distinct group within the *Trichoderma* genus. This cluster has moderate bootstrap support (65%), indicating some level of confidence in this relationship. JKJ18 is most likely a strain of *Trichoderma* species, potentially *Trichoderma koningiopsis* (Figure 2). The tree is rooted with *S. stipitis* (outgroup), which serves as a distant reference point to distinguish between the fungal genera. The bootstrap values indicate varying levels of confidence in different parts of the tree, with values above 70% generally considered reliable.

### 3.3. Tricalcium Phosphate (TCP) and Phytic Acid Calcium Salt (PCS) Solubilization

**Figure 3:**
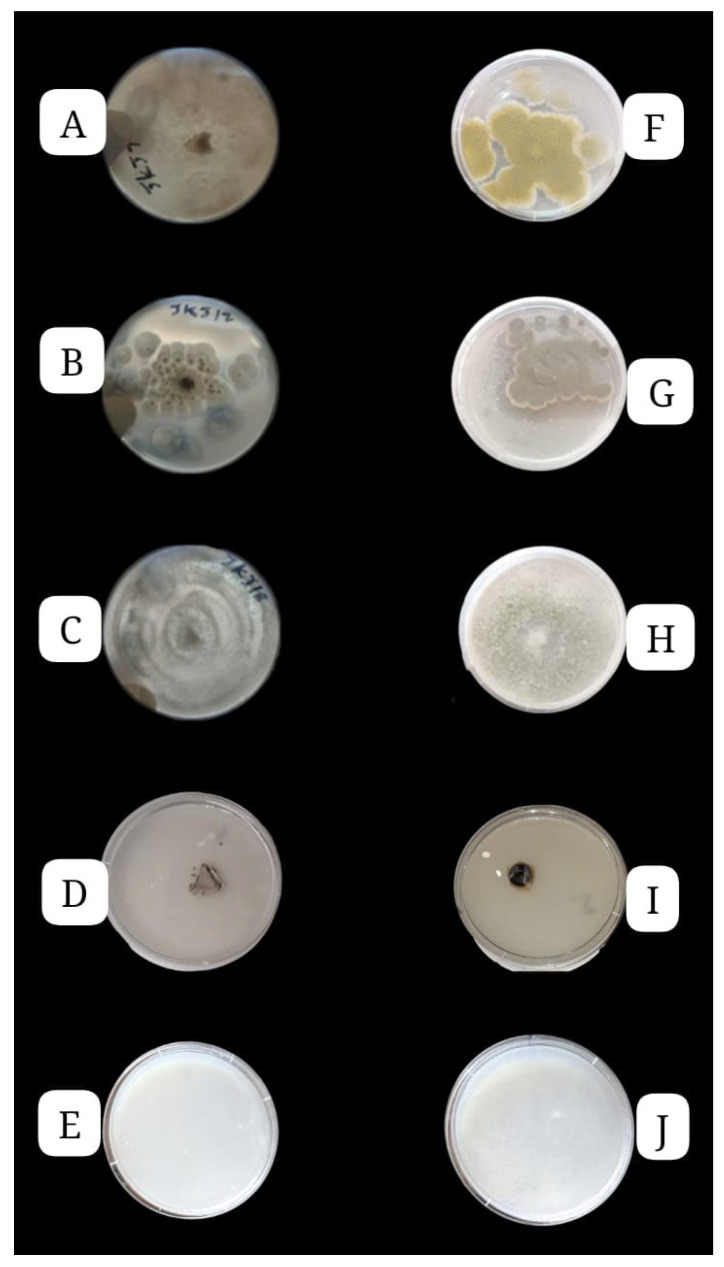
Solubilization of inorganic (**A**–**E**) and organic phosphate (**F**–**J**) by the fungal strains in NBRIP agar media supplemented with tricalcium phosphate (inorganic) (TCP) and phytic acid calcium salt (PCS) (organic) as an insoluble phosphate source after six days of culturing (*A. flavus* JKJ7 (**A**,**F**); *T. purpureogenus* JKJ12 (**B**,**G**); *T. koningiopsis* JKJ18 (**C**,**H**); uninoculated controls (**E**,**J**); no P solubilization (**D**,**I**)). Writings on plates (**A**–**C**) denotes strain numbers.

The three fungal strains, *Aspergillus flavus* JKJ7, *Talaromyces purpureogenus* JKJ12, and *Trichoderma koningiopsis* JKJ18, demonstrated the ability to solubilize phosphate. Figure 3 provides a visual representation of the fungal strains, illustrating varying degrees of growth typical of fungi, as well as the formation of clear zones on NBRIP media supplemented separately with tricalcium phosphate (TCP) and phytic acid calcium salt (PCS). While all three strains exhibited growth on both inorganic (TCP) and organic (PCS) phosphorus sources, they generally showed a stronger preference for the organic P source. Figure 3 compares the growth of the three phosphate-solubilizing fungi with Figure 3D and Figure 3I fungi, which exhibited poor growth on the NBRIP agar media supplemented with TCP and PCS, respectively. Additionally, Figure 3E and Figure 3J represent uninoculated controls to provide virtual aid in measuring the transformation of the inoculated media, which all have transformed drastically.

**Figure 4:**
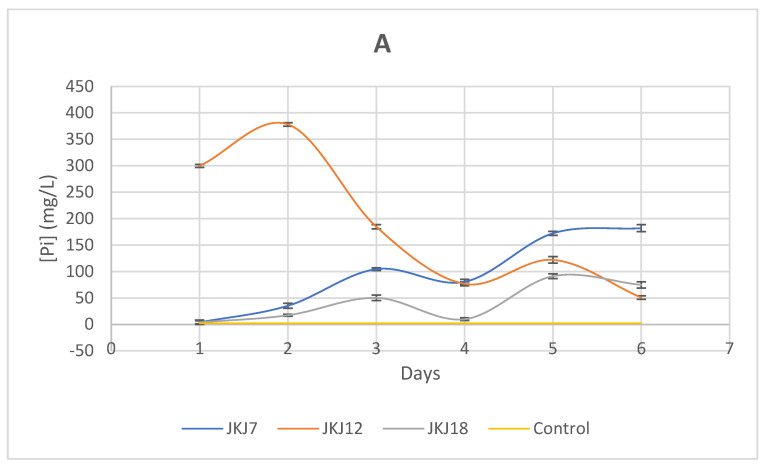

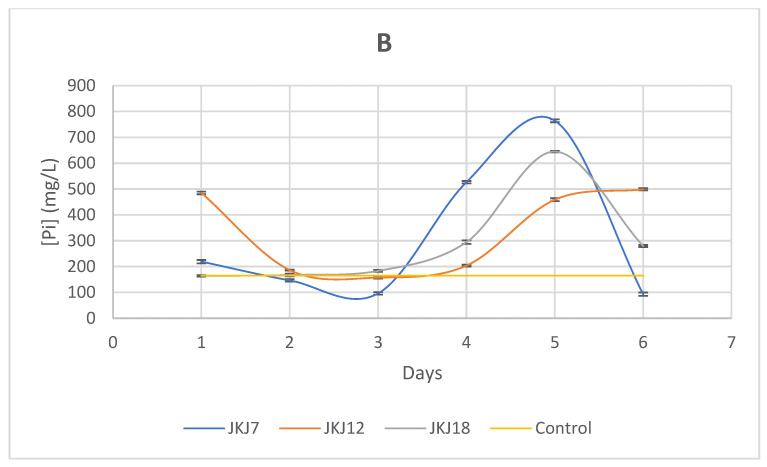
Solubilization of inorganic (**A**) and organic phosphate (**B**) by the fungal strains in NBRIP media supplemented with tricalcium phosphate (TCP) and phytic acid calcium salt (PCS) as an insoluble phosphate source for six days of culturing (JKJ7—*A. flavus*; JKJ12—*T. purpureogenus*; JKJ18—*T. koningiopsis*). The results are presented as a mean of three repeats and error bars of mean +/− SD.

A quantitative experiment was conducted where samples were collected daily for six consecutive days to measure the amount of soluble phosphate released into the medium when fungi were inoculated and compared with the uninoculated control. The data provided in Figure 4 showed variable amounts of soluble phosphate across the experimental period for all three fungal strains in broths with both TCP and PCS supplementation. In that six-day experimental period in TCP media, *T. purpureogenus* JKJ12 solubilized the highest amount of phosphate compared with the other strains, with the maximum soluble P of 378.14 mg/L recorded on day 2 of culturing. The fungal strain *A. flavus* JKJ7 exhibited moderate phosphate solubilization capability. The maximum amount of soluble P released was lower than that of *T. purpureogenus* JKJ12 but higher than *T. koningiopsis* JKJ18. *A. flavus* JKJ7 solubilized 181.98 mg/L P on day 6 of culturing while *T. koningiopsis* JKJ18 solubilized only 91.2 mg/L on day 5 (Figure 4A). In the broth supplemented with PCS, the fungal strain *A. flavus* JKJ7 had the highest concentration of soluble P with a maximum of 762.32 mg/L of P on day 5 of culturing. The second highest soluble P concentration was 644.78 mg/L, solubilized by *T. koningiopsis* JKJ18 on day 6 of culturing, while on day 4, *T. purpureogenus* JKJ 12 had its highest soluble P of 499.1 mg/L detected (Figure 4B).

### 3.4. Indole Acetic Acid Production (IAA)

**Figure 5:**
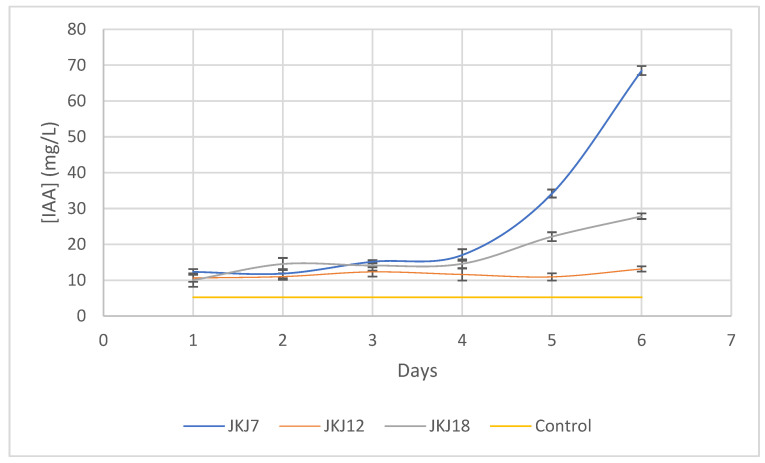
The production of auxin phytohormone (IAA) by the three fungal strains in YM broth supplemented with L-tryptophan (JKJ7—*A. flavus*; JKJ12—*T. purpureogenus*; JKJ18—*T. koningiopsis*). The results are presented as a mean of three repeats and error bars of mean +/− SD.

The three fungal strains, *A. flavus* JKJ7, *T. purpureogenus* JKJ 12, and *T. koningiopsis* JKJ18, produced detectable concentrations of IAA (Figure 5). The concentrations of IAA gradually increased from the day of first detection across all three fungal strains. The highest concentration of IAA produced was 68.51 mg/L, this concentration was produced by *A. flavus* JKJ7 on the sixth day of incubation. The fungal strains *T. koningiopsis* JKJ18 and *T. purpuregenus* JKJ12 produced the maximum concentration of IAA at 27.86 g/L and 13.15 mg/L, respectively, on the sixth day of incubation.

### 3.5. Siderophore Production

**Figure 6:**
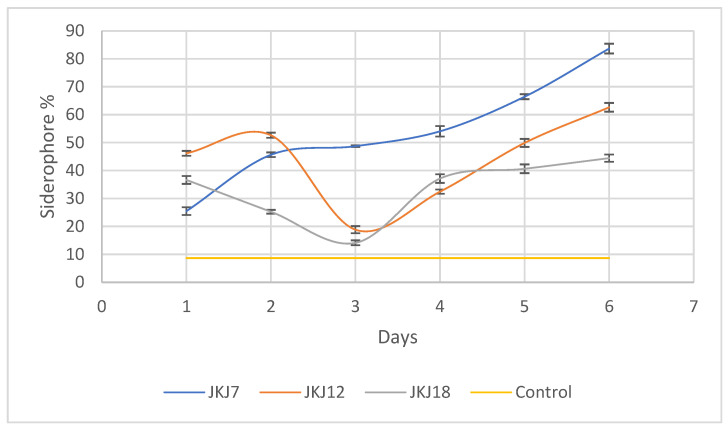
The production of iron-chelating molecules, siderophores, by the three selected fungal strains (JKJ7—*A. flavus*; JKJ12—*T. purpureogenus*; JKJ18—*T. koningiopsis*) in a vitamin-free media. The results are presented as a mean of three repeats and error bars of mean +/− SD.

The fungal strain *A. flavus* JKJ7 produced more siderophores than *T. purpureogenus* JKJ12 and *T. koningiopsis* JKJ18 (Figure 6). The three fungal strains had their highest siderophore production detected on day six. The fungal strain *A. flavus* JKJ7 is the highest producer at 83.7%, followed by *T. purpureogenus* JKJ12 at 62.7% and *T. koningiopsis* JKJ18 at 44.5%, respectively.

### 3.6. Principal Component Analysis (PCA)

**Figure 7:**
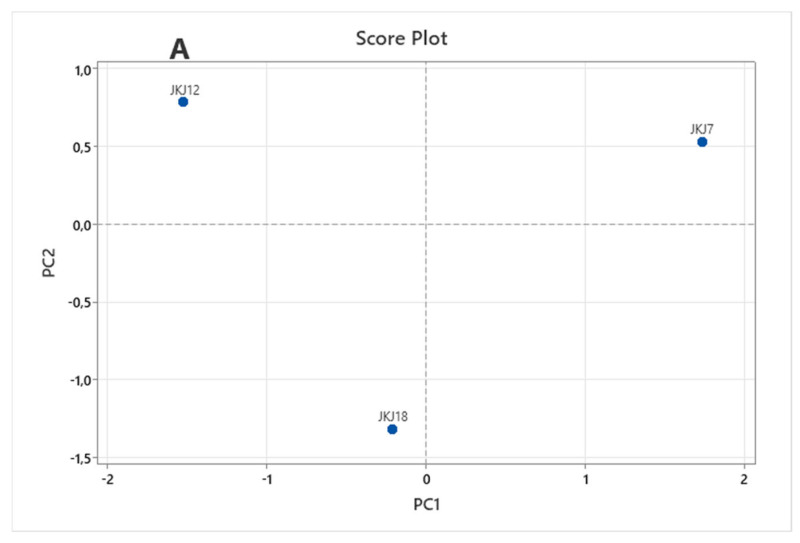

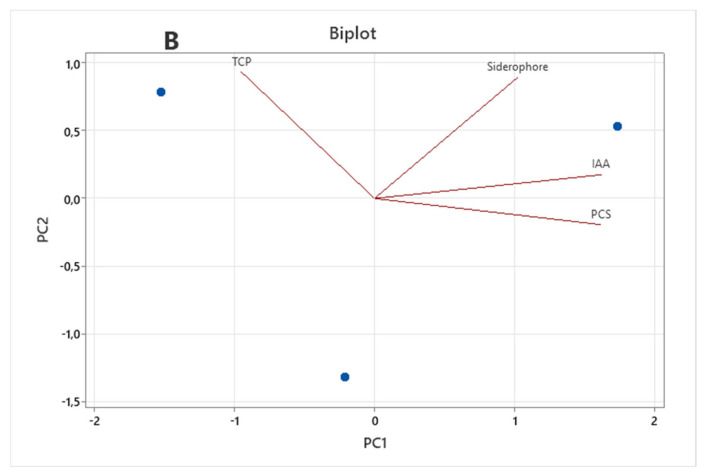
The Principal Component Analysis (PCA). Graph (**A**) shows a score plot of the two principal components obtained from the three fungal strains (JKJ7—*A. flavus*; JKJ12—*T. purpureogenus*; JKJ18—*T. koningiopsis*). Graph (**B**) is a biplot of the two principal components obtained from the four variables (TCP and PCS solubilization; IAA and siderophore production).

In the PCA plot shown in Figure 7, the score plot (Figure 7A) illustrates a wide distribution of the fungal strains, indicating significant variability among them. The biplot (Figure 7B) highlights the contributions of TCP solubilization, PCS solubilization, IAA production, and siderophore synthesis to the observed variability. These plots reveal that IAA production has the most significant impact on the separation of *A. flavus* JKJ7 from the other two strains, while TCP solubilization is the primary factor influencing the variability of *T. purpureogenus* JKJ12.

## 4. Discussion

Synthetic fertilizers, pesticides, and other chemical-based agricultural methods are frequently employed to boost crop productivity [34]. However, these synthetic products are often expensive for small-scale farmers, and improper management can harm non-target ecosystems [35,36]. In contrast, the use of microorganisms as biofertilizers presents a more economical and environmentally friendly alternative [37]. Microorganisms, particularly fungi, aid in the production of phytohormones, enhance nutrient uptake, and protect plants from stress, thus stimulating plant growth both directly and indirectly [28,38]. Given that non-bioavailable phosphorus (P) forms predominate in agricultural soils, fungi capable of converting these into bioavailable forms offer a sustainable approach to increasing crop productivity [31,39,40].

This study focused on three fungal strains—*Aspergillus flavus* JKJ7, *Talaromyces purpureogenus* JKJ12, and *Trichoderma koningiopsis* JKJ18—which demonstrated the ability to solubilize both organic (PCS) and inorganic (TCP) phosphates. All three strains exhibited typical fungal growth, with varying degrees of mycelial expansion and clear zone formation on NBRIP media. These observations indicate the ability of the strains to adapt to and utilize non-bioavailable phosphorus sources as sole phosphorus providers [41,42]. Phosphate solubilization in these strains is mediated either by organic acids (for TCP solubilization) or phytase activity (for PCS solubilization) [28,40,43].

*T. koningiopsis* Quantitative experiments revealed fluctuations in soluble phosphate levels (Pi), which reflect the dynamic nature of phosphate solubilization. Factors such as fungal growth stage, pH, nutrient availability, and enzymatic activity influence these fluctuations [31,44]. Among the strains, *T. purpureogenus* JKJ12 solubilized the highest amount of TCP *T. purpureogenus* (378.14 mg/L), while *A. flavus* JKJ7 solubilized the highest amount of PCS (762.32 mg/L). The solubilization capacity of JKJ18 was comparatively lower but still significant (91.2 mg/L from TCP and 644.78 mg/L from PCS). These findings are consistent with previous studies on similar fungal species and highlight the robust mechanisms for phosphate solubilization in these strains, indicating their potential in sustainable agriculture [28,40,43].

Principal Component Analysis (PCA) was applied to better understand the functional differences among the strains. The PCA results indicate that *A. flavus* JKJ7 was distinguished by its superior IAA production, while JKJ12 showed the highest phosphate solubilization ability with TCP. The distinct separation of the strains in the PCA plot reflects their individual strengths, suggesting that these fungi could be utilized based on specific soil nutrient conditions to maximize crop growth. This analysis helps reinforce the practical relevance of these strains for targeted agricultural applications, where selecting the most effective strain for particular soil conditions is key.

Soil fungi also play multiple roles in promoting plant growth beyond increasing nutrient bioavailability. They produce growth-promoting substances, such as auxins, gibberellins, siderophores, and cytokinin [28,45]. Specifically, the production of IAA and siderophores is crucial for promoting plant growth. IAA facilitates cell division and root elongation, while siderophores enhance iron uptake by forming iron–siderophore complexes in iron-deficient soils [46]. In this study, *A. flavus* JKJ7 produced the highest levels of IAA compared with the other two strains, highlighting its potential as a bioinoculant for enhancing root development. Similarly, *A. flavus* JKJ7 also exhibited the highest siderophore production, positioning it as the most promising strain for improving iron bioavailability in nutrient-poor soils. Previous studies have also demonstrated the effectiveness of IAA- and siderophore-producing fungi in promoting plant growth and competing with phytopathogens for essential nutrients like iron [44,47,48].

In a previous study, *T. purpureogenus* produced high levels of IAA (405 mg/L when supplemented with L-tryptophan), which correlated with its ability to promote wheat seedling growth under both normal and stressful conditions [49]. This highlights the importance of environmental adaptability for biofertilizers. Although fungal biofertilizers show great promise as sustainable alternatives or supplements to chemical fertilizers, their performance can be influenced by several environmental factors. Different geographical areas have unique environmental conditions that must be considered when developing biofertilizer formulations [50,51]. Therefore, researchers are continually isolating fungi from various regions to identify biofertilizers adapted to specific environments. To the best of our knowledge, this is the first study to isolate fungal biofertilizers from the biodiversity-rich soils of Sovenga Hill at the University of Limpopo. Given their adaptation to the local environmental conditions, these fungal strains are likely to perform optimally in regions with similar ecological characteristics.

## 5. Conclusions

In conclusion, this study demonstrated the potential of *Aspergillus flavus* JKJ7, *Talaromyces purpureogenus JKJ12*, and *Trichoderma koningiopsis* JKJ18 as biofertilizers due to their abilities to solubilize phosphate, produce indole-3-acetic acid (IAA), and synthesize siderophores. *T. purpureogenus* JKJ12 excelled in solubilizing inorganic phosphorus (TCP), while *A. flavus* JKJ7 was the most effective with organic phosphorus (PCS) and showed the highest production of IAA and siderophores. Principal Component Analysis (PCA) confirmed these functional differences, suggesting that the strains could be used individually or in combination to suit specific soil conditions. Future research, including field trials, will further validate their efficacy, with fungal consortia offering the potential to enhance biofertilizer performance. Overall, this study contributes to the advancement of fungal biofertilizers as sustainable alternatives to chemical fertilizers with the potential to improve nutrient availability, enhance plant growth, and support environmentally friendly agricultural practices.

## Figures and Tables

**Table 1:** Similarity between the sequence queried and the biological sequences within the NCBI database using Blast.

Sample Name	JKJ7	JKJ12	JKJ18
Predicated organism	*Aspergillus flavus*	*Talaromyces purpureogenus*	*Trichoderma koningiopsis*
Genbank Accession	MT645322.1	LT558947.1	MT102395.1
% Identity	100	100	99.66

## Data Availability

The raw data supporting the conclusions of this article will be made available by the authors on request.

## References

[1] Beltran-Pea A., Rosa L., D’Odorico P. (2020). Global food self-sufficiency in the 21st century under sustainable intensification of agriculture. Environ. Res. Lett..

[2] Shao Z., Mwakidoshi E.R., Muindi E.M., Soratto R.P., Ranjan S., Padhan S.R., Wamukota A.W., Sow S., Wasonga D.O., Nasar J. (2023). Synthetic Fertilizer Application Coupled with Bioslurry Optimizes Potato (*Solanum tuberosum*) Growth and Yield. Agronomy.

[3] Penuelas J., Coello F., Sardans J. (2023). A better use of fertilizers is needed for global food security and environmental sustainability. Agric. Food Secur..

[4] Andrino A., Guggenberger G., Kernchen S., Mikutta R., Sauheitl L., Boy J. (2021). Production of Organic Acids by Arbuscular Mycorrhizal Fungi and Their Contribution in the Mobilization of Phosphorus Bound to Iron Oxides. Front. Plant Sci..

[5] Peng Y., Huo W., Feng G. (2024). Maximising cotton phosphorus utilisation for zero surplus and high yields: A review of innovative P management strategies. Field Crops Res..

[6] Bedine M.A.B., Iacomi B., Tchameni S.N., Sameza M.L., Fekam F.B. (2022). Harnessing the phosphate-solubilizing ability of *Trichoderma* strains to improve plant growth, phosphorus uptake and photosynthetic pigment contents in common bean (*Phaseolus vulgaris*). Biocatal. Agric. Biotechnol..

[7] Devi R., Kaur T., Kour D., Yadav A., Yadav A.N., Suman A., Ahluwalia A.S., Saxena A.K. (2022). Minerals solubilizing and mobilizing microbiomes: A sustainable approach for managing minerals’ deficiency in agricultural soil. J. Appl. Microbiol..

[8] Lopez G., Ahmadi S.H., Amelung W., Athmann M., Ewert F., Gaiser T., Gocke M.I., Kautz T., Postma J., Rachmilevitch S. (2023). Nutrient deficiency effects on root architecture and root-to-shoot ratio in arable crops. Front. Plant Sci..

[9] Vassilev N., Mendes G.d.O. (2024). Soil Fungi in Sustainable Agriculture. Microorganisms.

[10] Shukla A.C. (2023). Entrepreneurship with Microorganisms.

[11] Jiang F., Zhang L., Zhou J., George T.S., Feng G. (2021). Arbuscular mycorrhizal fungi enhance mineralisation of organic phosphorus by carrying bacteria along their extraradical hyphae. New Phytol..

[12] Saranya K., Sundaramanickam A., Manupoori S., Kanth S.V. (2022). Screening of multi-faceted phosphate-solubilising bacterium from seagrass meadow and their plant growth promotion under saline stress condition. Microbiol. Res..

[13] Kumar V., Prasher I.B. (2023). Phosphate solubilization and indole-3-acetic acid (IAA) produced by *Colletotrichum gloeosporioides* and *Aspergillus fumigatus* strains isolated from the rhizosphere of *Dillenia indica* L. Folia Microbiol..

[14] Nagrale D.T., Chaurasia A., Kumar S., Gawande S.P., Hiremani N.S., Shankar R., Gokte-Narkhedkar N., Renu Y.G., Prasad Y.G. (2023). PGPR: The treasure of multifarious beneficial microorganisms for nutrient mobilization, pest biocontrol and plant growth promotion in field crops. World J. Microbiol. Biotechnol..

[15] Dev M., Kumar P., Singh S., Singh R. (2023). Role of Mutant Phosphate Solubilizing Microorganism on Growth of *Brassica juncea* (L.) cv RH 30. Indian J. Microbiol..

[16] Yang T., Li L., Wang B., Tian J., Shi F., Zhang S., Wu Z. (2022). Isolation, Mutagenesis, and Organic Acid Secretion of a Highly Efficient Phosphate-Solubilizing Fungus. Front. Microbiol..

[17] Brazhnikova Y.V., Shaposhnikov A.I., Sazanova A.L., Belimov A.A., Mukasheva T.D., Ignatova L.V. (2022). Phosphate Mobilization by Culturable Fungi and Their Capacity to Increase Soil P Availability and Promote Barley Growth. Curr. Microbiol..

[18] Parvez M., Hussain F., Khan M., Sajid H. (2023). Characterization of phosphate solubilizing fungal endophyte associated with roots of Coriandrum sativum L growing in water stressed soil. Symbiosis.

[19] Dhariwal A.G., Tarafdar J.C. (2023). A comparison of phytase efficiency originated from plant and fungal sources. GSC Biol. Pharm. Sci..

[20] Rico-Jiménez M., Muñoz-Mira S., Lomas-Martínez C., Krell T., Matilla M.A. (2023). Regulation of indole-3-acetic acid biosynthesis and consequences of auxin production deficiency in *Serratia* plymuthica. Microb. Biotechnol..

[21] Chen X., Smith S.M., Shabala S., Yu M. (2022). Phytohormones in plant responses to boron deficiency and toxicity. J. Exp. Bot..

[22] AKabir H., Bennetzen J.L. (2024). Molecular insights into the mutualism that induces iron deficiency tolerance in sorghum inoculated with *Trichoderma* harzianum. Microbiol. Res..

[23] Sultana S., Alam S., Karim M.M. (2021). Screening of siderophore-producing salt-tolerant rhizobacteria suitable for supporting plant growth in saline soils with iron limitation. J. Agric. Food Res..

[24] Sun Y., Wu J., Shang X., Xue L., Ji G., Chang S., Niu J., Emaneghemi B. (2022). Screening of Siderophore-Producing Bacteria and Their Effects on Promoting the Growth of Plants. Curr. Microbiol..

[25] El-Maraghy S.S., Tohamy A.T., Hussein K.A. (2021). Plant protection properties of the Plant Growth-Promoting Fungi (PGPF): Mechanisms and potentiality. Curr. Res. Environ. Appl. Mycol..

[26] Legodi L.M., La Grange D., Van Rensburg E.L.J., Ncube I. (2019). Isolation of Cellulose Degrading Fungi from Decaying Banana Pseudostem and Strelitzia alba. Enzym. Res..

[27] Makulana L., La Grange D.C., Moganedi K.L.M., Mert M.J., Phasha N.N., van Rensburg E.L.J. (2024). Screening and Isolation of Xylanolytic Filamentous Fungi from the Gut of Scarabaeidae Dung Beetles and Dung Beetle Larvae. Microorganisms.

[28] Sun X., Liu F., Jiang W., Zhang P., Zhao Z., Liu X., Shi Y., Sun Q. (2023). Talaromyces purpurogenus Isolated from Rhizosphere Soil of Maize Has Efficient Organic Phosphate-Mineralizing and Plant Growth-Promoting Abilities. Sustainability.

[29] Kiprotich K., Muoma J., Omayio D.O., Ndombi T.S., Wekesa C. (2023). Molecular Characterization and Mineralizing Potential of Phosphorus Solubilizing Bacteria Colonizing Common Bean (*Phaseolus vulgaris* L.) Rhizosphere in Western Kenya. Int. J. Microbiol..

[30] Jaya G.I., Utami S.N.H., Widada J., Yusuf W.A. (2020). Characterization of phosphate-solubilizing bacteria isolated from acidic Ultisol soil, South Borneo. IOP Conf. Ser. Earth Environ. Sci..

[31] Zhang X., Rajendran A., Grimm S., Sun X., Lin H., He R., Hu B. (2023). Screening of calcium- and iron-targeted phosphorus solubilizing fungi for agriculture production. Rhizosphere.

[32] Patel S., Prajapati V., Patel P. (2022). Isolation and Screening of Mineral Phosphate Solubilizing Microorganisms.

[33] Wang Y.-Y., Li P., Zhang B., Meng J., Gao Y., He X., Hu X. (2020). Identification of Phosphate-solubilizing Microorganisms and Determination of Their Phosphate-solubilizing Activity and Growth-promoting Capability. BioResources.

[34] Tang Q., Cotton A., Wei Z., Xia Y., Daniell T., Yan X. (2022). How does partial substitution of chemical fertiliser with organic forms increase sustainability of agricultural production?. Sci. Total Environ..

[35] Zainuddin N., Keni M.F., Ibrahim S.A.S., Masri M.M.M. (2022). Effect of integrated biofertilizers with chemical fertilizers on the oil palm growth and soil microbial diversity. Biocatal. Agric. Biotechnol..

[36] Sun B., Luo Y., Yang D., Yang J., Zhao Y., Zhang J. (2023). Coordinative Management of Soil Resources and Agricultural Farmland Environment for Food Security and Sustainable Development in China. Int. J. Environ. Res. Public Health.

[37] Balogun D.A., Oke M.A., Rocha-Meneses L., Fawole O.B., Omojasola P.F. (2022). Phosphate solubilization potential of indigenous rhizosphere fungi and their biofertilizer formulations. Agron. Res..

[38] Bessai S.A., Cruz J., Carril P., Melo J., Santana M.M., Mouazen A.M., Cruz C., Yadav A.N., Dias T., Nabti E.-H. (2023). The Plant Growth-Promoting Potential of Halotolerant Bacteria Is Not Phylogenetically Determined: Evidence from Two *Bacillus* megaterium Strains Isolated from Saline Soils Used to Grow Wheat. Microorganisms.

[39] Han M., Chen Y., Sun L., Yu M., Li R., Li S., Su J., Zhu B. (2023). Linking rhizosphere soil microbial activity and plant resource acquisition strategy. J. Ecol..

[40] Kong F., Ling X., Iqbal B., Zhou Z., Meng Y. (2023). Soil phosphorus availability and cotton growth affected by biochar addition under two phosphorus fertilizer levels. Arch. Agron. Soil Sci..

[41] Alam K., Barman M., Datta S.P., Annapurna K., Shukla L. (2023). Modification of Inorganic Fractions of Phosphorus by Phosphate-Solubilising Microorganisms in Conjunction with Phosphorus Fertilisation in a Tropical Inceptisol. J. Soil Sci. Plant Nutr..

[42] Alikhani H.A., Beheshti M., Pourbabaee A.A., Etesami H., Rahmani H.A., Noroozi M. (2023). Phosphorus Use Management in Paddy Fields by Enriching Periphyton with Its Phosphate-Solubilizing Bacteria and Fungi at the Late Stage of Rice Growth. J. Soil Sci. Plant Nutr..

[43] Bononi L., Chiaramonte J.B., Pansa C.C., Moitinho M.A., Melo I.S. (2020). Phosphorus-solubilizing *Trichoderma* spp. from Amazon soils improve soybean plant growth. Sci. Rep..

[44] Prasad A., Dixit M., Meena S.K., Suman, Kumar A. (2021). Qualitative and quantitative estimation for phosphate solubilizing ability of *Trichoderma* isolates: A natural soil health enhancer. Mater. Today Proc..

[45] Tandon A., Fatima T., Anshu, Shukla D., Tripathi P., Srivastava S., Singh P.C. (2020). Phosphate solubilization by *Trichoderma koningiopsis* (NBRI-PR5) under abiotic stress conditions. J. King Saud. Univ. Sci..

[46] Ahmad A., Moin S.F., Liaqat I., Saleem S., Muhammad F., Mujahid T., Zafar U. (2023). Isolation, Solubilization of Inorganic Phosphate, and Production of Organic Acids by Individual and Co-inoculated Microorganisms. Geomicrobiol. J..

[47] Behera B.C., Yadav H., Singh S., Mishra R., Sethi B., Dutta S., Thatoi H. (2017). Phosphate solubilization and acid phosphatase activity of *Serratia* sp. isolated from mangrove soil of Mahanadi river delta, Odisha, India. J. Genet. Eng. Biotechnol..

[48] Iqbal Z., Ahmad M., Raza M.A., Hilger T., Rasche F. (2024). Phosphate-Solubilizing *Bacillus* sp. Modulate Soil Exoenzyme Activities and Improve Wheat Growth. Microb. Ecol..

[49] Zhou G., Fan K., Li G., Gao S., Chang D., Liang T., Li S., Liang H., Zhang J., Che Z. (2023). Synergistic effects of diazotrophs and arbuscular mycorrhizal fungi on soil biological nitrogen fixation after three decades of fertilization. iMeta.

[50] Eshboev F. (2020). The Use of Three Fungal Strains in Producing of Indole-3-Acetic Acid and Gibberelllic Acid. https://www.researchgate.net/publication/343892528.

[51] Mohamed A.H., El-Megeed F.H.A., Hassanein N.M., Youseif S.H., Farag P.F., Saleh S.A., Abdel-Wahab B.A., Alsuhaibani A.M., Helmy Y.A., Abdel-Azeem A.M. (2022). Native Rhizospheric and Endophytic Fungi as Sustainable Sources of Plant Growth Promoting Traits to Improve Wheat Growth under Low Nitrogen Input. J. Fungi.

